# Strengthening United Nations country support on non-communicable diseases and mental health: an analysis of United Nations Sustainable Development Cooperation Frameworks

**DOI:** 10.3389/fpubh.2025.1741538

**Published:** 2026-01-13

**Authors:** Scott Chiossi, Ilaria Corazza, Roy Small, Alexey Kulikov, Nicholas Banatvala

**Affiliations:** 1 Secretariat of the United Nations Inter-Agency Task Force on the Prevention and Control of NCDs, World Health Organization, Geneva, Switzerland; 2 United Nations Development Programme, New York, NY, United States

**Keywords:** Cooperation Frameworks, mental health, non-communicable diseases, Sustainable Development Goals, United Nations

## Abstract

Non-communicable diseases (NCDs) and mental health conditions are among the major development challenges of the 21st century. Their impact across the 2030 Agenda calls for a coordinated United Nations (UN) response. UN Sustainable Development Cooperation Frameworks (UNSDCFs) facilitate UN joint action by serving as strategic planning tools co-designed by the UN country team and government. This paper describes how NCDs and mental health are included in these frameworks by reviewing UNSDCF documents using a key term search and categorising the extracted content by intervention area. The results show that compared to the 2012–2015 period, when 34% of UNSDCFs included NCDs, there has been progress in their prioritisation. Of the 114 UNSDCFs launched between 2020 and 2024, 69% included NCDs and 75% included mental health. The lowest levels of NCD integration were found in the World Health Organization African, Eastern Mediterranean, and Americas regions, while the African and Western Pacific regions had the lowest levels including mental health. UNSDCFs referencing NCDs and mental health often did so broadly and were missing specific indicators and outputs, leading to reduced clarity of what is expected by the UN system and weaker accountability. NCD risk factors were found to be generally absent in UNSDCFs. These findings highlight the growing recognition of NCDs and mental health on the development agenda, yet their integration in UNSDCFs is often inadequate and requires a more synergistic and targeted approach that can be translated into whole-of-UN system action at country level.

## 1 Introduction

Non-communicable diseases (NCDs) are responsible for seven out of 10 deaths globally, primarily from cardiovascular diseases, cancers, chronic respiratory diseases and diabetes (1). One in eight people in the world live with a mental disorder, anxiety and depressive disorders being the most common (2). NCDs and mental health conditions affect multiple Sustainable Development Goals (SDGs): reduced earning potential and out-of-pocket medical expenses drive families into poverty (SDG 1); caregiving responsibilities limit education and employment prospects, particularly in girls and women (SDG 4 and SDG 5); absence from work and lower productivity reduce economic growth (SDG 8); and poorer populations facing greater exposure to NCD risk factors exacerbate inequalities between and within countries (SDG 10) (3, 4).

Recognising NCDs as a major development challenge, United Nations (UN) Member States in 2011 called on UN agencies to support national multisectoral NCD responses and in 2014 committed to integrate NCD measures into United Nations Development Assistance Frameworks (UNDAFs), now known as United Nations Sustainable Development Cooperation Frameworks (UNSDCFs) (5, 6). UNSDCFs are “the most important instrument for the planning and implementation of United Nations development activities in each country […]” (7). A UNSDCF is co-designed by the UN country team (UNCT) and government concerned, detailing development priorities to jointly drive forward over a three to 5 year period (8). These frameworks have been at the centre of UN system reform, originating in 1997 to enhance UN programmatic coherence at country level, and revitalised in 2018 to better position the UN system in supporting countries implement the 2030 Agenda (9, 10).

Amid tightening resources, in 2025 the UN embarked on a new wave of restructuring under the UN80 initiative (11). At the same time, Member States requested during the fourth high-level meeting of the UN General Assembly (UNGA) on NCDs, mental health and well-being for UN agencies to scale up and mobilise coordinated support (12). This makes UNSDCFs ever more relevant as a tool to foster greater UN efficiency, yet there is no detailed overview to refer to on how NCDs and mental health are being integrated in these frameworks. To support the effective use of UNSDCFs in scaling up joint UN NCD and mental health action, this paper describes progress in the prioritisation of these conditions across regions and how to leverage these tools to strengthen UN coordination and accountability.

## 2 Reviewing how NCDs and mental health are included in UN Cooperation Frameworks

Following the first high-level meeting on NCDs in 2011, the United Nations Development Programme (UNDP) and World Health Organization (WHO), including the Secretariat of the UN Interagency Task Force on the Prevention and Control of NCDs (hereafter Task Force), disseminated guidance to encourage UNCTs to mainstream NCDs into their UNSDCFs (13). The Task Force was established in 2013 to bring together UN agencies to support countries build a multisectoral NCD response and has since monitored and reported on the inclusion of NCDs and mental health in UNSDCFs globally. This paper builds on the methodology developed by the Task Force to not only determine if UNSDCFs include NCDs and mental health, but also how they are reflected (14).

A total of 114 UNSDCFs rolled out between 2020 and 2024 were included in this analysis. 2020 was selected as the starting year as it marked the transition from UNDAFs to UNSDCFs and 2024 was used as the cutoff to ensure all UNSDCFs were available when conducting the analysis in 2025. UNSDCFs were identified by searching the UN Sustainable Development Group’s (UNSDG) “Countries and Territories” webpage (15), which provides UNSDCFs’ start and end dates and full documents. If a UNSDCF document was not available online, it was obtained from the WHO country office. Two UNSDCFs were multi-country, covering 14 countries and territories in the Pacific and 21 in the Caribbean. This brought the total number of countries and territories covered in this analysis to 147. Multi-country UNSDCFs were treated as subregional frameworks and not broken down by country or territory. The full list of UNSDCFs analysed and their implementation period is shown in Supplementary Table 1.

To identify NCD and mental health content in UNSDCFs, key words were searched using the documents’ search function. Key words were selected based on the main types of conditions, major risk factors and priority intervention areas described in WHO NCDs and mental health global action plans (16, 17). UNSDCFs were available in English, French, Spanish or Portuguese. To standardise the search across documents, key words were translated from English into the three additional languages and checked against WHO official terminology. For selected terms, abbreviations and a partial word search was used to ensure comprehensive coverage and capture derivatives. Following the Task Force methodology, NCD and mental health content was only considered if part of the UNSDCFs’ strategic priorities and/or results matrix sections. Strategic priorities describe national development priorities, guiding the UNCT in planning action. The results matrix includes outcomes, outputs and indicators to monitor progress and report results (8, 14). Key words are listed in Supplementary Table 2 and the inclusion and exclusion criteria for NCD and mental health content are described in Supplementary Boxes 1, 2.

UNSDCF content addressing NCDs and mental health was extracted into a spreadsheet and coded using the categories in Table 1. In line with the targets of the WHO Global Action Plan on NCDs, NCD content was categorised by primary risk factors and disease groups. Mental health content was classified according to the focus areas of the Mental Health Action Plan 2013–2030 global targets, which primarily reflect system-level issues rather than specific conditions. These plans have been created through global and regional consultations and their targets endorsed by WHO Member States. The coding categories derived from these plans were reviewed and agreed upon by the authors.

**Table 1:** Framework for categorising NCD and mental health content extracted from UNSDCFs.

Content type	Categories
UNSDCF information	WHO Region Country name (or subregion if multi-country) Start year End year Content extracted Page number Strategic priorities or results matrix section Priority, indicator, output or outcome NCD and/or mental health (NCD, MH, NCD&MH)
NCDS	Air pollution Alcohol consumption Physical inactivity Tobacco use Unhealthy diet Cancer Cardiovascular diseases Chronic respiratory diseases Diabetes NCDs (as a general thematic area) Other
Mental health	Community-based mental health Data collection and research Integration of mental health into primary care Mental health (as a general thematic area) Policy and legislation Provision of mental health services and psycho-social support Suicide Other

Extracted content was divided between two authors for coding, taking into account their familiarity with the UNSDCF’s language. To ensure consistency, a subset of the coded content was cross-checked by both authors. Where the content did not fit into any of the pre-set categories, it was categorised as “Other” and tagged with a relevant theme. If an additional recurring theme emerged, a specific category was created. Extracted content that addressed several themes was coded under multiple categories.

## 3 Global progress in prioritizing NCDs and mental health

No country is untouched by the social and economic impacts of NCDs and mental health conditions (5). Their disproportionate burden on low- and middle-income countries (LMICs), cyclical relationship with poverty, and synergies with multiple SDGs, make these conditions a key strategic focus for UNSDCFs (4, 13, 18).

**Figure 1:**
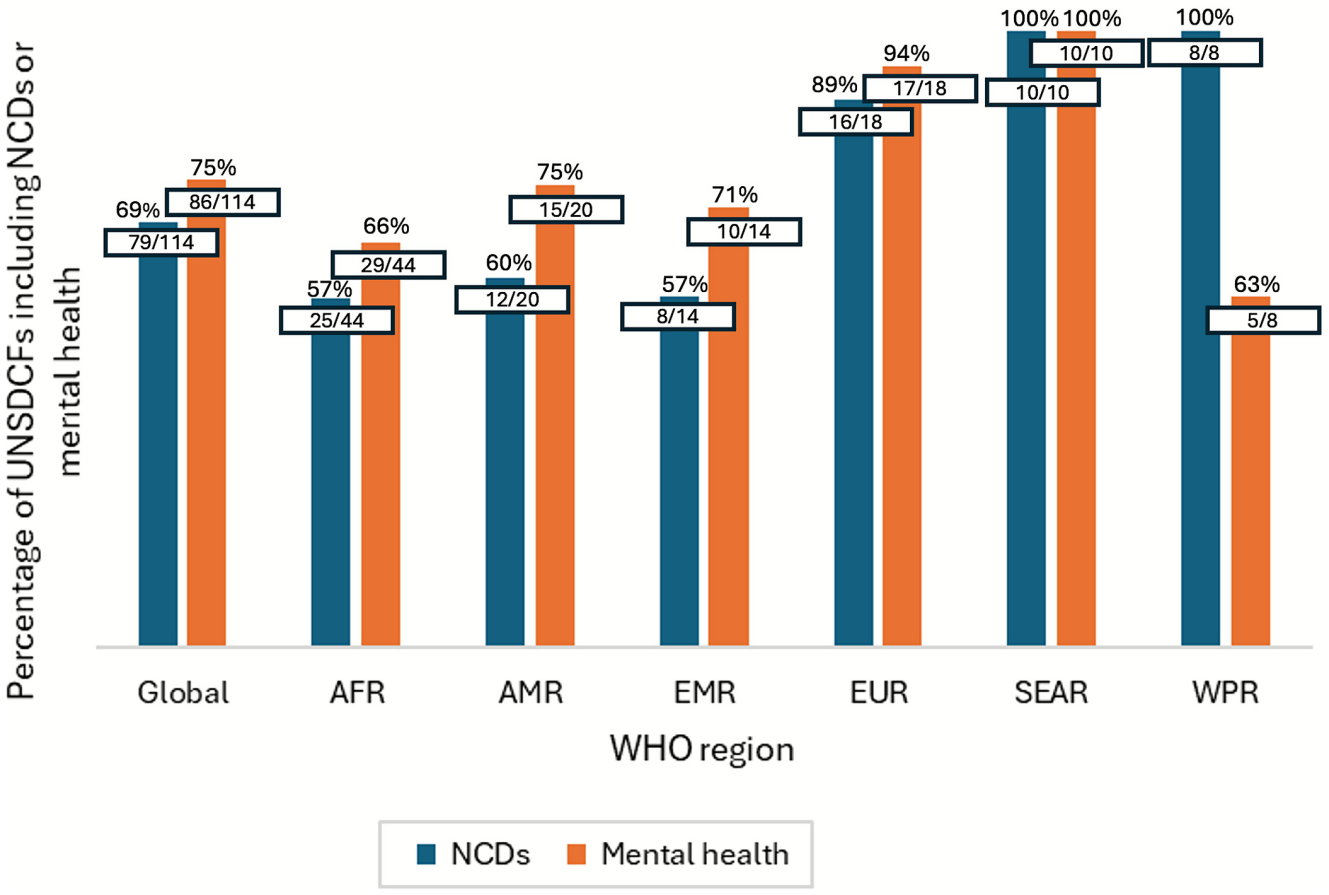
Percentage of UNSDCFs including NCDs and mental health by WHO region. AFR, African Region; AMR, Region of the Americas; EMR, Eastern Mediterranean Region; EUR, European Region; SEAR, South-East Asia Region; WPR, Western Pacific Region.

Since the first review of UNSDCFs launched between 2012 and 2015, when only 34% (31/90) of UNSDCFs included NCDs (and mental health was not yet reviewed), there has been progress in their prioritisation (14). Among the 114 UNSDCFs launched between 2020–2024, 69% (79/114) integrated NCDs and 75% (86/114) mental health, including the Caribbean and Pacific multi-country UNSDCFs (Figure 1). Yet, a gap remains of 35 countries not reflecting NCDs and 28 not reflecting mental health.

The WHO African (57%, 25/44), Eastern Mediterranean (57%, 8/14) and Americas (60%, 12/20) regions had the lowest percentage of UNSDCFs that included NCDs, while the African (66%, 29/44) and Western Pacific (63%, 5/8) regions had the lowest for mental health. This may be considered low coverage given the 15% or higher probability of dying prematurely from NCDs between the ages of 30 and 70 in the majority of countries in these regions (19).

Although this analysis does not demonstrate a direct link, it may be that the increase in NCD inclusion in UNSDCFs was partly influenced by the momentum built from the 2011, 2014, and 2018 UN high-level meetings as well as the Task Force efforts to engage UNCTs on integrating these conditions in national development plans (20). Sustaining this progress will likely require overcoming ongoing barriers to NCD and mental health prioritisation, such as lack of data on their burden, constrained national capacity, industry interference, competing priorities in development assistance, and chronic underfunding (21–25). In addition, research on the impact of integrating NCDs and mental health into broader development programmes and funding streams could provide further insights into their cross-cutting benefits across the SDGs (4).

## 4 Governments’ and UN country teams’ selection of NCDs and mental health focus areas

The UN system is not expected to address every development issue in a country (8) and UNSDCFs should not prioritise every NCD, mental health condition, and associated risk factors, but rather identify together with the government which represent the highest burden and where the UN can offer clear added value (13). Table 2 provides the number of UNSDCFs referencing specific risk factors, NCDs, mental health intervention areas, which are further disaggregated by WHO region in Supplementary Table 3.

**Table 2:** UNSDCFs referencing specific NCD and mental health areas (absolute and percentage values).

Thematic group	NCD and mental health areas addressed by UNSDCFs	Count of UNSDCFs	Percentage of UNSDCFs (*N* = 114)
NCD risk factors	Overweight and/or obesity	28	25%
	Unhealthy diet	13	11%
	Tobacco	11	10%
	Alcohol	10	9%
	Air pollution	10	9%
	Hypertension	8	7%
	Physical inactivity	6	5%
NCDs	NCDs (as a general thematic area)	65	57%
	Cancer	28	25%
	Diabetes	24	21%
	Cardiovascular diseases	20	18%
	Chronic respiratory diseases	20	18%
Mental health	Victims of emotional and/or psychological violence	55	48%
	Provision of mental health services and psycho-social support	39	34%
	Mental health (as a general thematic area)	36	32%
	Mental health of young people and children	29	25%
	Mental health service provision to victims of violence	20	18%
	Migrant and/or refugee mental health	5	4%
	Community-based mental health	4	4%
	Policy and legislation	4	4%
	Suicide	4	4%
	Right to mental and physical health	4	4%
	Integration of mental health into primary care	3	3%
	Data collection and research	2	2%
	Trauma counselling	2	2%

In the reviewed UNSDCFs, NCD risk factors were generally absent despite their high burden across regions. Only six (5%) UNSDCFs considered physical inactivity even though nearly 1.8 billion adults worldwide are at risk of disease due to sedentary lifestyle (26). Just 10 (9%) UNSDCFs considered alcohol even though high consumption is recorded in most WHO regions, namely across the Americas and Europe (27). Only 10 (9%) UNSDCFs specified air pollution as a health issue and yet there are 7 million annual deaths related to air pollution, 85% of which are linked to NCDs (1). Tobacco was only included in 11 (10%) UNSDCFs, despite 21% of adults aged 15 years and older globally use tobacco, surpassing more than 30% of adults in multiple countries in the WHO European and South-East Asia regions (1, 19). Notably, overweight and obesity were considered in 28 (25%) UNSDCFs, with 15 of these focusing on malnutrition in children in line with SDG indicator 2.2.2.

Including these risk factors in UNSDCFs can have implications beyond the NCD response. Indeed, their low representation is a potential missed opportunity as a system-wide UN approach can help address the multisectoral nature and significant socioeconomic impact of these risk factors (13). For example, alcohol is not only an NCD risk factor but also affects road safety, youth development, and violence, falling within mandates of agencies such as UNDP, UNICEF, United Nations Population Fund (UNFPA), and the United Nations Office on Drugs and Crime (UNODC) (28). A joint approach can minimise duplication of efforts, improve data collection and information sharing, enhance coordinated engagement across ministries and other partners, and leverage multiple policy levers (29).

When it came to NCDs, cancer was mentioned most frequently, appearing in 25% (28/114) of UNSDCFs, followed by diabetes (21%), cardiovascular diseases (18%), and chronic respiratory diseases (18%). These diseases were primarily reflected with the inclusion of SDG indicator 3.4.1 in the results matrix. Six UNSDCFs specified breast and/or cervical cancer. Cervical cancer is a clear example of a disease that benefits from action by multiple UN agencies, including support from UNICEF on Human Papillomavirus vaccination, UNFPA and WHO to strengthen screening and management, and the Joint United Nations Programme on HIV/AIDS (UNAIDS) for integrating cervical cancer care in HIV services. Countries with high burden of cervical cancer such as in sub-Saharan Africa, Central America and South-East Asia are likely to benefit from a UN joint approach to deliver prevention and care practices (30, 31).

Delivering mental health and psychosocial services also requires coordinated efforts and specialised expertise to effectively reach vulnerable groups (32). Numerous UNSDCFs included provision of mental health services and psychosocial support (34%; 39/114) or mentioned mental health broadly (32%; 36/114). Victims of emotional and psychological violence were often referenced across UNSDCFs (48%; 55/114) due to the frequent inclusion of SDG indicators 16.1.3, 16.2.1, and 5.2.1, which measure populations affected by psychological violence, among other forms of violence. Mental health in young people and children was included in a quarter of UNSDCFs, often under SDG indicators 16.2.1 and 4.2.1 (25%; 29/114).

Suicide was among the least covered categories, found in 4% (4/114) of UNSDCFs. This highlights a major gap as more than 720,000 people die due to suicide each year, over 70% of these occur in LMICs, and represent the third leading cause of death among people aged 15 to 29 (33). Mental health of migrants and refugees was also only mentioned in 4% (5/114) of UNSDCFs, despite the 123 million forcibly displaced people as of 2024 (34). With LMICs hosting over 70% of refugees and their difficulty in accessing health services (34, 35), UNSDCFs could provide a potential entry point for strengthening coordinated action among relevant specialised agencies to deliver mental health and psychosocial support to these vulnerable groups.

## 5 Leveraging UN Cooperation Frameworks for joint planning and accountability

**Figure 2:**
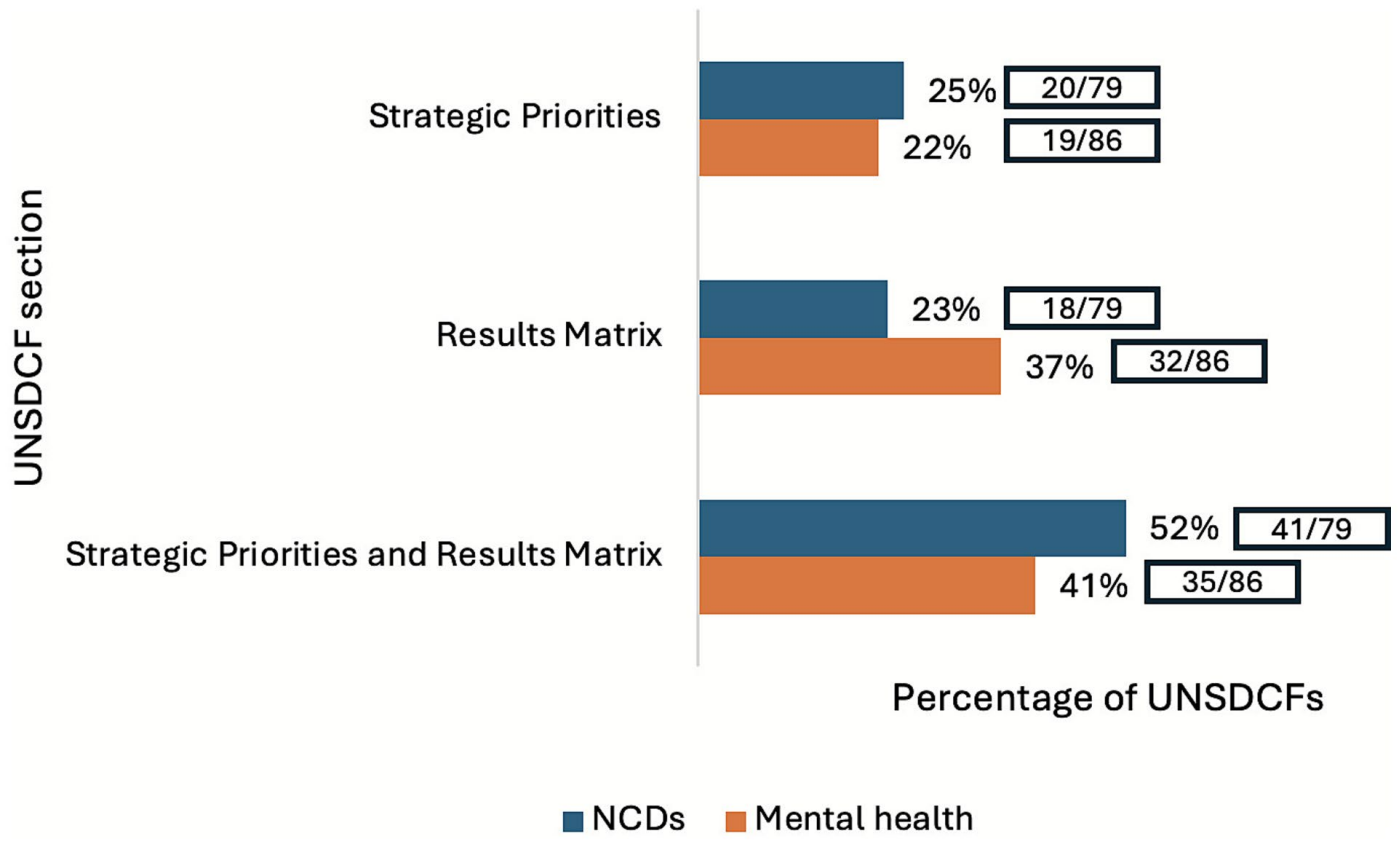
Percentage of UNSDCFs that include NCDs and mental health in the results matrix and/or strategic priorities sections.

UNSDCFs are expected to be anchored in government priorities and serve as tools to hold the UN accountable (7, 8). To ensure NCDs and mental health are comprehensively included in UNSDCFs, they should be reflected in both the strategic priorities and results matrix sections (13). However, only 52% (41/79) of UNSDCFs reflected NCDs and 41% (35/86) mental health in both sections (Figure 2).

Reflecting NCDs and mental health in the strategic priorities section positions them as key development issues, which in turn can be incorporated in UN joint workplans and programmes to strengthen inter-agency collaboration (8). However, UNSDCFs that include NCDs and mental health often do so generally and as part of a long list of health concerns, diluting their prioritisation. Only 51% (31/61) of UNSDCFs’ strategic priorities specified a targeted condition and/or intervention for NCDs and 69% (37/54) for mental health. This broad framing leads to agency-specific country plans to superficially align with UNSDCFs, rather than directly deriving from them as recommended (36).

Moreover, having specific NCD and mental health outputs and indicators in the results matrix can help monitor progress and strengthen accountability. Around 48% (55/114) of UNSDCFs integrated NCD indicators and 57% (65/114) included mental health indicators. Only 3% (3/114) of UNSDCFs had an outcome that referenced NCDs and 9% (10/114) included it as an output. No UNSDCF had a mental health outcome and 8% (9/114) included it as an output. While outcomes describe broader system changes and may not often specify NCDs and mental health, outputs are integral to describing concrete activities that are directly attributable to the UN system. Lack of NCD and mental health outputs may lead to reduced accountability and clarity of what is expected by the UN system.

**Figure 3:**
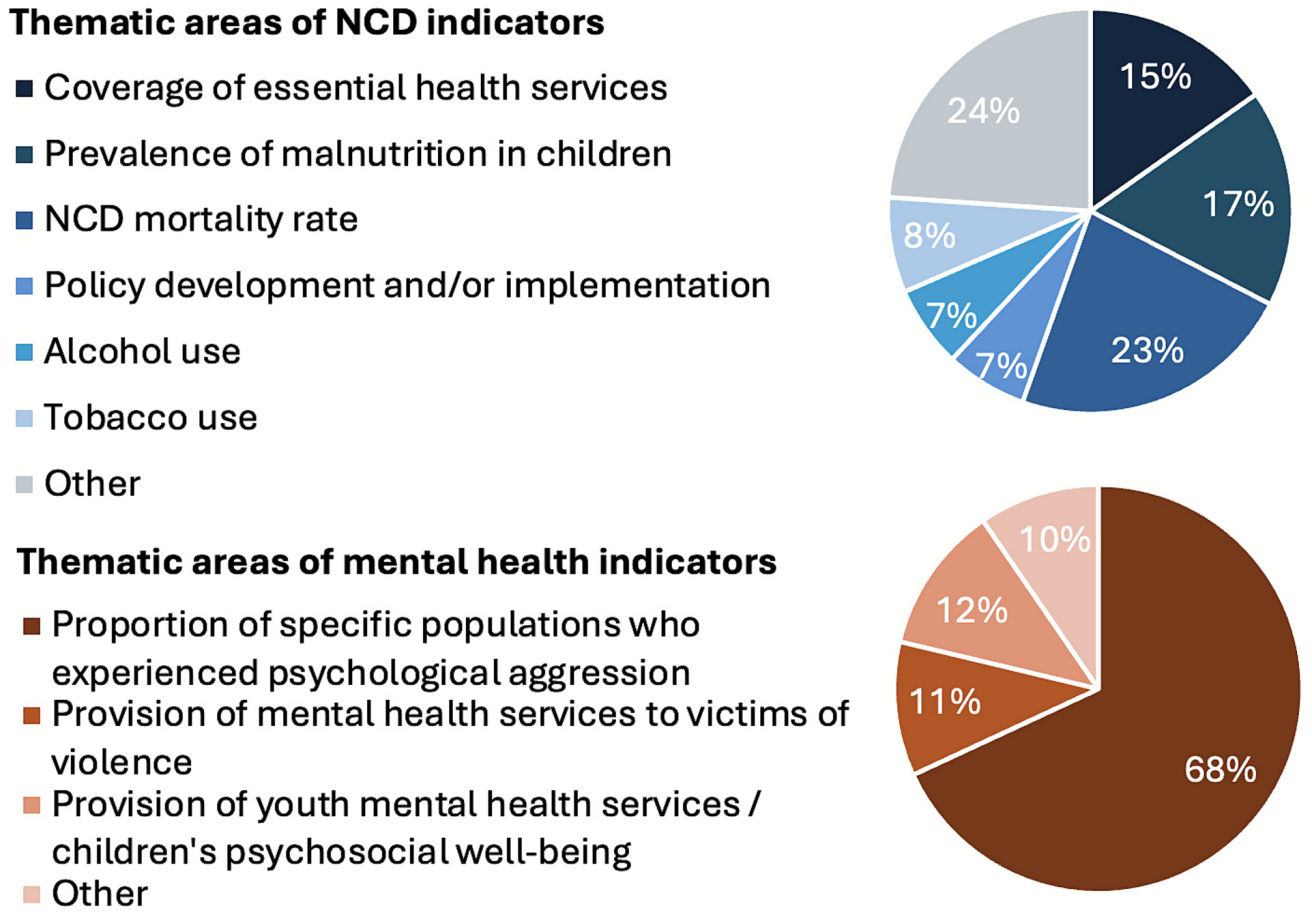
Distribution of NCD and mental health indicators by thematic area.

From the UNSDCFs analysed, 92 NCD- and 94 mental health related-indicators were extracted, most of which were linked to SDG indicators (Figure 3). For NCDs, 23% (21/92) measured NCD mortality rate (linked to SDG indicator 3.4.1), 17% (16/92) measured prevalence of malnutrition, including overweight, in children (linked to SDG indicator 2.2.2), and 15% (14/92) measured coverage of essential health services (linked to SDG indicator 3.8.1). Six indicators were for alcohol use, including three SDG indicator 3.5.2, and seven for tobacco use, including five SDG indicator 3.a.1. For mental health, a large majority (68%, 64/94) of indicators were linked to victims of psychological violence (SDG indicators 16.1.3, 16.2.1, and 5.2.1). Only two UNSDCFs included SDG indicator 3.4.2 on suicide mortality and two for SDG indicator 3.9.1 on mortality attributed to air pollution.

The UNSDCF guidance developed by UNSDG and the guidance on NCDs integration in UNDAFs developed by WHO, UNDP, and the Task Force, are useful tools that can help overcome some of the gaps and barriers identified in this paper (8, 13). Box 1 outlines examples of steps to build a stronger argument for integrating NCDs and mental health in UNSDCFs. These efforts may be facilitated by the UN Resident Coordinator’s office as a leading entity in the UNSDCF development process, WHO through technical support and health data, and UNDP bringing expertise in governance, economics and a multidimensional approach to the SDGs. The Task Force UNDAF guidance as well as Task Force UN Agency Briefs provide further insights into the specific roles and responsibilities of different UN agencies in supporting NCD and mental health action (37). Continuous advocacy for the prioritisation of NCDs and mental health in UNSDCFs is also needed from UN agencies and civil society at global and regional level. The Task Force, WHO and UNDP, in collaboration with the UN Sustainable Development Group, could support this process by updating existing UNSDCF guidance, organising information sessions for Resident Coordinators and UNCTs, and continuously monitoring progress to ensure accountability.

Box 1 Approaches to strengthening the integration of NCDs and mental health into UN Cooperation Frameworks.

- Collect national data to demonstrate the social and economic burden of NCDs and mental health conditions, using tools such as NCD and mental health investment cases and “STEPS” survey for NCD risk factor data.
- Reflect these data in the UN common country analysis (UN CCA), that is an assessment of countries’ progress and gaps, and the foundation to determining UNSDCF priorities.
- Involve NCD and mental health civil society organisations in the multistakeholder consultations of the UNSDCF development process, serving as advocates and bringing perspectives from the community, including marginalised populations.
- Align NCDs and mental health action with broader national development priorities by identifying focus areas and target groups from existing government policies and setting out the co-benefits of an integrated approach.
- Engage UN agencies with technical expertise on the identified focus areas from the start to ensure ownership, clarity of roles and responsibilities, and coordinated planning.
- Ensure strategic priorities are matched with dedicated outputs and indicators to strengthen accountability. Align these with SDG indicators to help address SDG data gaps and develop comprehensive country situational analysis.

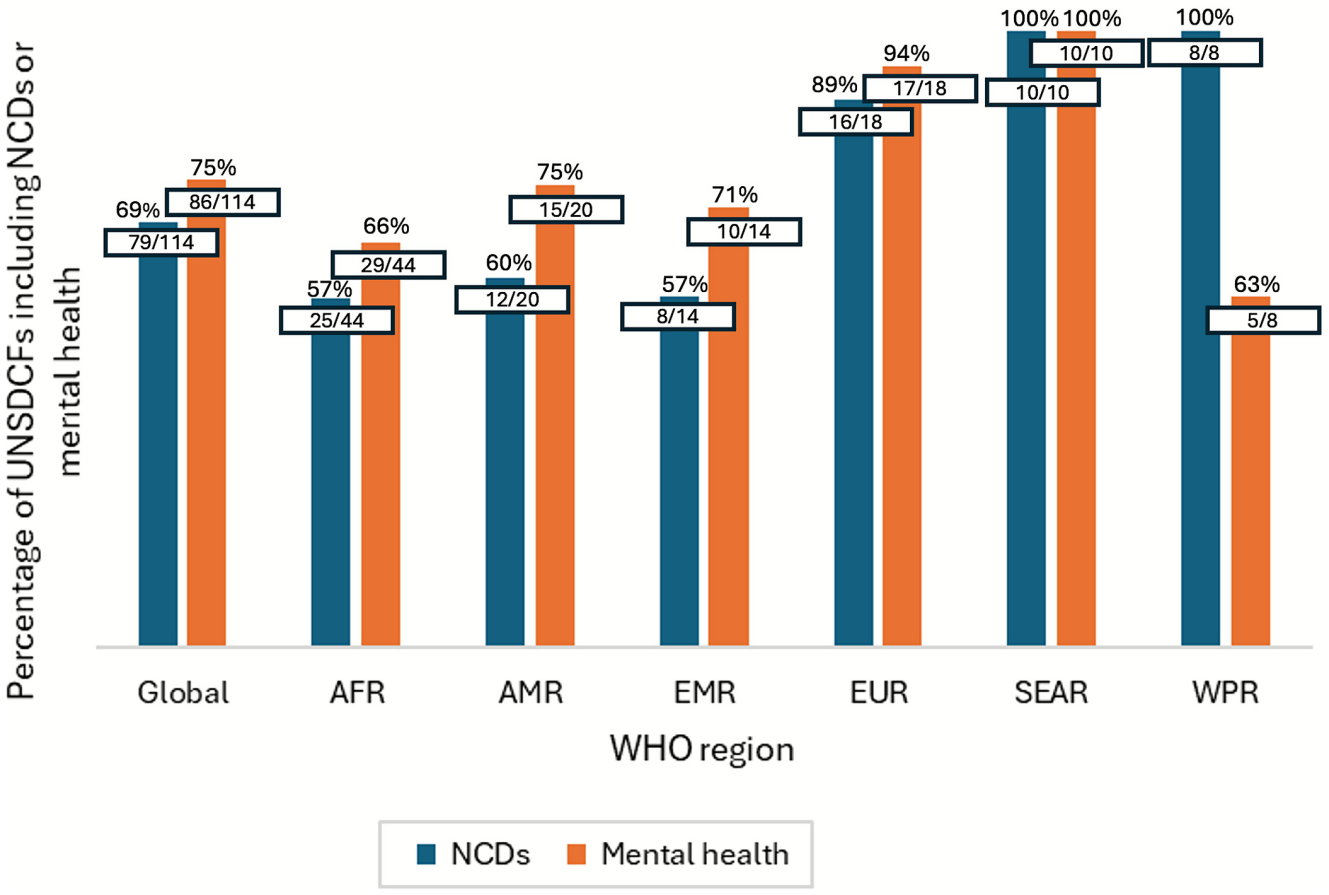

## 6 Overcoming fragmentation to unlock the full potential of a UN system-wide response

UNSDCFs aim to increase UN efficiency by facilitating joint UN planning and programming, acting as a valuable tool for fostering collaboration, aligning financial frameworks and reducing duplication of efforts. However, systemic UN governance and coordination challenges can act as a barrier to their implementation. This is reflected in the limited uptake of joint activities, making up less than one quarter of UNSDCFs’ budgets (8, 38).

The 2025 UN system-wide evaluation on progress towards a new generation of UNCTs reported weak collective ownership of UNSDCFs. Existing inter-agency competition over resources, funding and, to a lesser extent, mandates remains a key barrier. To maximise synergies, there is a need for greater transparency in work planning and resource mobilisation within UNCTs, backed by effective leadership from Resident Coordinators. Governments are encouraged to provide stronger oversight and hold UNCTs to higher standards, their active engagement in and ownership of UNSDCFs incentivises greater alignment of UN agencies’ programmes (39).

In line with the UN Funding Compact, the use of inter-agency pooled funding can also drive joint UN action. One example is the Heatlh4Life Fund, established by UNDP, UNICEF, and WHO, providing catalytic support to LMICs to scale up NCD and mental health action (40). However, contributions to inter-agency pooled funds remain low, and require overcoming structural financing flaws, including reliance on a small set of donors and preference for earmarked contributions (36, 39). The need to overcome fragmentation in development cooperation has been recognised by Heads of State and Government at the Fourth International Conference on Financing for Development, with commitments to prioritise financial support to countries though multilateral institutions and interagency pooled funds (41). The UN system can at the same time conduct joint resource mobilisation, strengthen accountability, and increase visibility of the impact of their work (42).

Existing joint UN NCD and mental health initiatives can serve as practical models for expanding or building new joint country programmes. For example, the Tobacco-Free Farms initiative brings together the Food and Agriculture Organization (FAO), World Food Programme (WFP), WHO and others to help farmers shift from tobacco growing to alternative food crops (43). In Uganda and Nepal, the WHO-led “SAFER” initiative supported by UNDP and the Task Force Secretariat is helping governments implement a package of interventions to reduce alcohol consumption (44). The International Development Law Organization (IDLO) and WHO joined forces under “RECAP” to assist countries implement regulatory and fiscal measures that promote healthy diets and physical activity (45). UNICEF and WHO are working together in 13 countries on addressing mental health and psychosocial well-being and development of children and adolescents (46).

Other examples of country-led initiatives include the Thailand UN Thematic Working Group on NCDs, which successfully convened stakeholders from non-health sectors (47), Project BRAVE in the Philippines, a collaboration between WHO, UNICEF, and UNFPA, on addressing mental health and gender-based violence (48), and in Iraq, where UNDP and WHO supported the Government in conducting psychosocial support trainings for the reintegration of displaced individuals (49). Under the Task Force’s WHO-UNDP Joint Programme to catalyse country action for NCDs and mental health governance and investment, nine country offices worked in close partnership to support governments advance multisectoral action plans, coordination mechanisms, legislations, and investment cases (25).

## 7 Limitations and future research

There are limitations to the analysis described in this paper. The list of key terms used to search UNSDCFs was not exhaustive and certain NCD, mental health conditions or intervention areas may have been missed. Translation of key terms may have also resulted in overlooking terminology specific to the different languages analysed. Coding categories were not externally validated and broader themes pertinent to NCDs and mental health (e.g., health system strengthening and social services) were not considered unless explicitly linked to these conditions. UNSDCFs rolled out between 2020 and 2024 may have been missed if they were not reported on the UNSDG website.

It is important to note that the exclusion of NCDs and mental health from UNSDCFs does not imply that national UN joint activities are not ongoing. Future studies may consider reviewing UN Country Team Result Reports and conducting interviews with national stakeholders to obtain information into existing initiatives. This could also help obtain clarity on factors influencing prioritisation of NCDs and mental health in UNSDCFs, including the availability of funding and sociopolitical dynamics driving the decision-making process, which may in turn explain variations between countries and regions. Obtaining direct insights into the current barriers and enablers to the UNSDCF development process could help inform revisions of UNSDCF guidance documents. Finally, it would be of interest to explore how the Task Force and the UN high-level meetings have contributed to the uptake of NCDs and mental health in UNSDCFs over the past decade.

## 8 Conclusion

UNCTs can better assist Member States in reaching the SDGs and scaling up their NCD and mental health response by strengthening coordination across agencies and addressing these conditions through system-wide UN action. This is especially important in the current context of UN reform, for which UNSDCFs can serve as a key tool to increase UN efficiency. Although the inclusion of NCDs and mental health in UNSDCFs has increased over the years, their integration could be strengthened through targeted strategies that can be translated into UN joint programmes and clear outputs and indicators to reinforce accountability. No UN agency can tackle the complex development challenges of NCDs and mental health conditions alone. With only 5 years remaining to achieving the SDGs it is critical to accelerate action through a unified approach.
