# Supplementary material for: Strengthening United Nations country support on non-communicable diseases and mental health: an analysis of United Nations Sustainable Development Cooperation Frameworks
Source: Front Public Health. 2026 Jan 13;13:1741538. doi: 10.3389/fpubh.2025.1741538 (PMC12835282; doi:10.3389/fpubh.2025.1741538)
Supplement: Supplementary file 1 [file Data_Sheet_1.pdf]

Supplementary Material

Supplementary Table 1. Inclusion of NCDs and mental health in UNSDCFs rolled out between 2020 and 2024.

Country/Territory	UNSDCF implementation period	NCDs included	Mental health included
African Region			
Algeria	2023-2027	Yes	Yes
Angola	2024-2028	Yes	No
Benin	2023-2026	Yes	Yes
Botswana	2022-2026	Yes	Yes
Burkina Faso	2023-2025	Yes	Yes
Burundi	2023-2027	No	No
Cabo Verde	2023-2027	No	No
Cameroon	2022-2026	No	Yes
Central African Republic	2023-2027	No	Yes
Chad	2024-2026	No	No
Comoros	2022-2026	No	Yes
Congo	2020-2024	No	Yes
Côte d'Ivoire	2021-2025	Yes	Yes
Democratic Republic of the Congo	2020-2024	No	Yes
Equatorial Guinea	2024-2028	Yes	Yes
Eritrea	2022-2026	Yes	No
Eswatini	2021-2025	Yes	Yes
Ethiopia	2020-2025	No	Yes
Gabon	2023-2027	No	No
Ghana	2023-2025	Yes	No
Guinea	2024-2028	No	Yes
Guinea-Bissau	2022-2026	No	No
Kenya	2022-2026	Yes	Yes
Lesotho	2024-2028	Yes	Yes
Liberia	2020-2024	No	Yes
Madagascar	2024-2028	No	Yes
Malawi	2024-2028	Yes	Yes
Mali	2020-2024	No	No
Mauritania	2024-2027	Yes	No
Mauritius	2024-2028	Yes	No
Mozambique	2022-2026	No	Yes
Niger	2023-2027	Yes	Yes

Nigeria	2023-2027	Yes	Yes
Sao Tome and Principe	2023-2027	Yes	Yes
Senegal	2024-2028	Yes	Yes
Somalia	2021-2025	Yes	No
South Africa	2020-2025	Yes	Yes
South Sudan	2023-2025	No	Yes
The Gambia	2024-2028	No	No
Togo	2023-2027	No	Yes
Uganda	2021-2025	Yes	No
United Republic of Tanzania	2022-2027	Yes	Yes
Zambia	2023-2027	Yes	No
Zimbabwe	2022-2026	Yes	Yes
Americas Region			
Argentina	2021-2025	Yes	Yes
Bolivia (Plurinational State of)	2023-2027	Yes	Yes
Brazil	2023-2027	No	No
Chile	2023-2026	No	Yes
English- and Dutch-speaking Caribbean (Multi-Country UNSDCF)	2022-2026	Yes	Yes
Colombia	2024-2027	No	No
Costa Rica	2023-2027	Yes	Yes
Cuba	2020-2024	Yes	No
Dominican Republic	2023-2027	No	Yes
Ecuador	2022-2026	Yes	Yes
El Salvador	2022-2026	No	Yes
Guatemala	2020-2025	Yes	Yes
Haiti	2023-2027	No	No
Honduras	2022-2026	Yes	Yes
Mexico	2020-2025	No	Yes
Panama	2021-2025	Yes	Yes
Paraguay	2020-2024	Yes	No
Peru	2022-2026	No	Yes
Uruguay	2021-2025	Yes	Yes
Venezuela (Bolivarian Republic of)	2023-2026	Yes	Yes
Eastern Mediterranean Region			
Afghanistan	2023-2025	No	Yes
Djibouti	2022-2024	No	No
Egypt	2023-2027	Yes	Yes
Iran (Islamic Republic of)	2023-2027	Yes	Yes
Iraq	2020-2024	No	No
Lebanon	2023-2025	Yes	Yes
Libya	2023-2025	Yes	Yes
Morocco	2023-2027	Yes	Yes

Pakistan	2023-2027	Yes	Yes
Saudi Arabia	2022-2026	No	No
occupied Palestinian territory, including east Jerusalem	2023-2025	No	Yes
Syrian Arab Republic	2022-2024	No	Yes
Tunisia	2021-2025	Yes	No
Yemen	2022-2024	Yes	Yes
European Region			
Albania	2022-2026	Yes	Yes
Armenia	2021-2025	Yes	Yes
Azerbaijan	2021-2025	Yes	Yes
Belarus	2021-2025	Yes	Yes
Bosnia and Herzegovina	2021-2025	Yes	Yes
Georgia	2021-2025	Yes	Yes
Kazakhstan	2021-2025	Yes	Yes
Kosovo	2021-2025	Yes	Yes
Kyrgyzstan	2023-2027	Yes	Yes
Montenegro	2023-2027	Yes	Yes
North Macedonia	2021-2025	Yes	Yes
Republic of Moldova	2023-2027	No	Yes
Serbia	2021-2025	Yes	Yes
Tajikistan	2023-2026	Yes	No
Türkiye	2021-2025	Yes	Yes
Turkmenistan	2021-2025	Yes	Yes
Ukraine	2022-2023	No	Yes
Uzbekistan	2021-2025	Yes	Yes
South-East Asia Region			
Bangladesh	2022-2026	Yes	Yes
Bhutan	2024-2028	Yes	Yes
Cambodia	2024-2028	Yes	Yes
India	2023-2027	Yes	Yes
Indonesia	2021-2025	Yes	Yes
Maldives	2022-2026	Yes	Yes
Nepal	2023-2027	Yes	Yes
Sri Lanka	2023-2027	Yes	Yes
Thailand	2022-2026	Yes	Yes
Timor-Leste	2021-2025	Yes	Yes
Western Pacific Region			
China	2021-2025	Yes	Yes
Lao People's Democratic Republic	2022-2026	Yes	No
Malaysia	2021-2025	Yes	No
Mongolia	2023-2027	Yes	Yes
Pacific (Multi-Country UNSDCF)	2023-2027	Yes	Yes

Papua New Guinea	2024-2028	Yes	Yes
Philippines	2024-2028	Yes	Yes
Viet Nam	2022-2026	Yes	No

Supplementary Table 2. Key terms searched in UNSDCFs.

**NCD key terms**	• Air pol*<br>• Alcohol<br>• Asthma<br>• Blood pressure<br>• Cancer<br>• Card*<br>• Chronic<br>• CVD<br>• Diab*<br>• Diet<br>• Fat*<br>• Glucose<br>• Heart attack<br>• Hyperten*<br>• Medic*<br>• NCD<br>• Non-com*<br>• Noncom*<br>• Obes*<br>• Overweight<br>• Physical<br>• Respir*<br>• Salt<br>• Smok*<br>• Sodium<br>• Sport<br>• Stroke<br>• Sugar<br>• Therapy<br>• Tobacco<br>• Treatment<br>• Risk factors

**Mental health key terms**	• Anx*<br>• Anorexia<br>• Autis*<br>• Behaviour<br>• Bipolar<br>• Bulimia<br>• Cognitive<br>• Counselling<br>• Dementia<br>• Depression<br>• Disorder<br>• Eating disorder<br>• Mental<br>• Neur*<br>• Post-traumatic stress<br>• PTSD<br>• Psych*<br>• Schizophrenia<br>• Therap*<br>• Suicid*

Supplementary Box 1. Inclusion and exclusion criteria for NCD content.

Inclusion criteria

The UNSDCF includes the following as a priority, indicator, outcome, or output:

- NCD morbidity and mortality
- Specific national health system response to NCDs
- Tobacco use, physical inactivity, alcohol consumption, unhealthy diets, and air pollution as a health risk factor
- Biological risk factors, including raised blood pressure, diabetes and obesity
- NCD and communicable disease comorbidities

Exclusion criteria

- Chronic diseases that are not NCDs (when referring to "chronic disease" it must clearly indicate NCDs)
- Indicators that measure morbidity/mortality but do not specify if the morbidity/mortality is due to an NCD (e.g. neonatal mortality rate (deaths per 1,000 live births))
- Indicators that measure a health coverage or response in general and do not specify NCDs
- Malnutrition that focuses on undernourishment or does not specify measurement of overweight and/or obese individuals
- The UNSDCF only mentions the burden of NCDs in the context analysis

Supplementary Box 2. Inclusion and exclusion criteria for mental health content.

Inclusion criteria

The UNSDCF includes the following as a priority, indicator, outcome, or output:

- Mental health conditions
- Mental health services
- Mental health promotion and prevention programmes
- Integration of mental health in primary health care
- Community-based mental health facilities
- Mental health laws, policies, and plans
- Suicide
- Mental health in the context of emergencies/disasters
- Individuals receiving counselling/therapy (children, prisoners, elderly etc.)
- Mental health condition and communicable disease comorbidities
- Individuals who suffered from psychological violence
- Individuals who suffered from physical, sexual and gender-based violence and received mental health services

Exclusion criteria

- Social development programmes that do not specify mental health
- Substance use disorders
- The UNSDCF only mentions the burden of mental health in the context analysis

Supplementary Table 3. UNSDCFs referencing specific NCD and mental health areas by WHO region. AFR = African Region; AMR = Region of the Americas; EMR = Eastern Mediterranean Region; EUR = European Region; SEAR = South-East Asia Region; WPR = Western Pacific Region.

	NCD and mental health areas addressed by UNSDCFs	Count of UNSDCFs	Percentage of UNSDCFs (N=114)	Count of UNSDCF per WHO Region					
				AFR	AMR	EMR	EUR	SEAR	WPR
**NCD risk factors**	Overweight and/or obesity	28	25%	6	6	2	5	6	3
	Unhealthy diet	13	11%	5	1	1	2	3	1
	Tobacco	11	10%		1	1	4	5	
	Alcohol	10	9%	1			5	4	
	Air pollution	10	9%	1		1	3	3	2
	Hypertension	8	7%	4	1	1		1	1
	Physical inactivity	6	5%	1		1	2	1	1
**NCDs**	NCDs (as a general thematic area)	65	57%	23	5	8	14	8	7
	Cancer	28	25%	8	2	4	9	2	3
	Diabetes	24	21%	6	3	3	8	3	1
	Cardiovascular diseases	20	18%	4	2	3	8	2	1
	Chronic respiratory diseases	20	18%	5	2	2	8	2	1
**Mental health**	Victims of emotional and/or psychological violence	55	48%	17	11	4	15	5	3
	Provision of mental health services and psycho-social support	39	34%	12	4	8	6	7	2
	Mental health (as a general thematic area)	36	32%	7	7	8	6	5	3
	Mental health of young people and children	29	25%	6	2	4	9	6	2
	Mental health service provision to victims of violence	20	18%	10		4	4	1	1
	Migrant and/or refugee mental health	5	4%	1	2		2		

	Community-based mental health	4	4%			1		3	
	Policy and legislation	4	4%		2	2			
	Suicide	4	4%		1	1	2		
	Right to mental and physical health	4	4%	2	1		1		
	Integration of mental health into primary care	3	3%	1		2			
	Data collection and research	2	2%				1		1
	Trauma counselling	2	2%	2					
